# Determinants Affecting the Rationing of Nursing Care and Professional Burnout among Oncology Nurses

**DOI:** 10.3390/ijerph19127180

**Published:** 2022-06-11

**Authors:** Aneta Piotrowska, Aleksandra Lisowska, Iwona Twardak, Karolina Włostowska, Izabella Uchmanowicz, Eleonora Mess

**Affiliations:** 1 Department of Hematology and Transplantation, Lower Silesian Oncology, Pulmonology and Hematology Center, 53-439 Wroclaw, Poland; anetapiotrowska2020@wp.pl; 2 Department of Nursing and Obstetrics, Wroclaw Medical University, 51-618 Wroclaw, Poland; aleksandra.lisowska@umw.edu.pl (A.L.); iwona.twardak@umw.edu.pl (I.T.); eleonora.mess@umw.edu.pl (E.M.); 3 Department of Cancer Prevention, Faculty of Health Sciences, Medical University of Warsaw, 02-091 Warsaw, Poland; karolina.wlost@gmail.com

**Keywords:** oncology nurses, rationing of nursing care, missed nursing care, professional burnout syndrome, life satisfaction, job satisfaction

## Abstract

Rationing of nursing care (RNC) is characterized by the omission of any aspect of the required patient care, resulting in incomplete or delayed nursing activities. Oncology nurses are exposed to a very high psychological burden, which can lead to the development of professional burnout syndrome (PBS). The level of PBS might be related to life and job satisfaction. This study aimed to identify determinants affecting RNC and reveal the relationship between RNC, life and job satisfaction, and the PBS levels among oncology nurses. The sample was a hundred oncology nurses from four hospitals in Poland with a mean age of 43.26 ± 10.69 years. The study was conducted from March 2019 to February 2020. The self-administered sociodemographic questionnaire and validated scales determining missed nursing care, job and life satisfaction, and life orientation were used: Basel Extent of Rationing of Nursing Care-Revised (BERNCA-R), Satisfaction with Job Scale (SWJS), Satisfaction with Life Scale (SWLS), Life Orientation Test-Revised (LOT-R), and Maslach Burnout Inventory (MBI). The mean BERNCA score was 1.55 ± 0.15, which indicates the frequency of RNC was between “never” and “rarely”. The mean SWJS score was 11.71 ± 5.97, which showed that nurses were “dissatisfied” and “rather dissatisfied” with their job. A low SWLS score was reported by 59% of nurses, which means that more than half of the respondents described their life satisfaction as low. In LOT-R, 66% of nurses reported pessimistic and 31% neutral life orientation. The mean overall MBI score was 49.27 ± 19.76 points (EE = 63.56 ± 25.37, DEP = 37.2 ± 24.95, and lack of PA = 47.05 ± 22.04), which means that half of the nurses perceived burnout and half did not. Additionally, the higher the job satisfaction (SWJS), the more frequent the RNC (BERNCA) (*p* < 0.05). The greater the EE, the stronger the sense of lack of PA, and the higher the PBS (MBI) level, the less frequent the RNC (BERNCA). In conclusion, there is a phenomenon of omission of some aspects of care among oncology nurses, but it is not frequent and concerns areas not directly related to therapeutic tasks, but requiring effort and not resulting in quick noticeable effects. It depends only little on life satisfaction and more on job satisfaction and PBS level. The results may indicate the professionalism of Polish nurses, their responsibility towards their patients’ life and health, and the sense of mission that enables them to perform their duties regardless of the external and internal difficulties. The presence of the PBS phenomenon in oncology nurses highlights the need for continued research in this area.

## 1. Introduction

Rationing of nursing care (RNC) and missed nursing care (MNC) are terms that have been used for several years around the world with regard to the work of nurses. The term RNC was first used in 2006 by American nurse Beatrice J. Kalisch [1] to refer to the total or partial omission of any aspect of required patient care, resulting in incomplete or delayed nursing activities. RNC may result in patient dissatisfaction or deterioration. Nurses are responsible for the quality of care, so identifying omissions and factors associated with omissions is an important component in taking action to restructure nursing services [2].

RNC has been recognized based on the observations of the work of U.S. nurses, but it applies to all countries worldwide, regardless of the health care system’s organization or funding [3]. Most evidence of RNC comes from nursing questionnaires or the opinions of the patients. Delayed or unfinished nursing care involves all aspects of clinical, emotional, or administrative care. RNC is both economically challenging (care is delivered within a socioeconomic framework) and ethically challenging (requires decisions that potentially conflict with one’s personal and professional values) [4].

The model of missed nursing care, developed by Kalisch et al. [1], considered the structural factors that contributed to missed care. These factors include work resources, material resources, teamwork, and communication. Currently, it is believed that staffing shortages and impaired team communication are the main factors contributing to RNC [5]. There are also factors related to the work environment: workplace organization, allocation of human resources, material resources, and organizational support [6,7].

The growing phenomenon of professional burnout syndrome (PBS) is associated with changes in civilization and increasing professional demands. Work-related stress is the cause of dissatisfaction with work, chronic fatigue, and emotional exhaustion [8]. Christina Maslach [9] developed the most popular definition of PBS as “a syndrome of emotional exhaustion, depersonalization, and a diminished sense of personal accomplishment that can occur in people who work with others in some specific way”.

There are many causes of PBS, but they can be grouped into three main areas: factors related to personality structure (age, gender, neurotic tendencies, extraversion), specifics of interpersonal relations (relations with patients, emotional involvement, relations with superiors, competition, conflicts, communication disturbances, mobbing), and organizational factors (staff shortages, poor equipment, night work, noise, stress, and constant pressure) [10,11].

PBS manifests on three levels: physical, emotional, and mental. On the physical level, it includes chronic weakness, fatigue, headaches, muscle aches, increased risk of infection, sleep disturbances, changes in eating habits, and a tendency to take excessive amounts of drugs or stimulants [12]. Emotional symptoms manifest by increasing apathy, a sense of disillusionment, a lack of desire to act, alienation, a tendency to cry, and a lack of prospects [13]. Finally, the psychological aspects of PBS include the loss of self-esteem, negative attitude toward oneself and one’s surroundings, cynicism, aggression, non-acceptance, and disregard for others.

Oncology nurses perform various professional activities and participate in specialized treatment modalities including oncological surgery, chemotherapy, and radiotherapy. Oncology is commonly thought of as a field associated with pain and death and extremely difficult emotions (uncertainty, fear, anger, fright) [14]. The multitasking nature of the work of oncology nurses is associated with a very high psychological load, and these nurses are exposed to stress and exhibit PBS symptoms. When the psychological load is accompanied by low social prestige, disproportionate salaries, improper relations at work, and organizational errors, disappointment, mental, and physical exhaustion lead to full-blown PBS [15].

This study aimed to identify the determinants affecting RNC and examine the relationship between the RNC, life and job satisfaction, and PBS levels among oncology nurses. The following two specific aims were provided: (1) to identify the determinants of care rationing and levels of job and life satisfaction among nurses, and (2) to assess the relationship of care rationing with levels of PBS and life and job satisfaction. Additionally, four research hypotheses were constructed: (1) High levels of job and life satisfaction reduce the PBS level among oncology nurses; (2) job seniority and type are among the factors determining the PBS level, and there are some nurses who show symptoms of PBS; (3) rationing of care depends on the nurses’ personal feelings in the sphere of job and life satisfaction and the PBS level; and (4) from the perspective of the risk of PBS among oncological nurses, it is necessary to develop and implement methods aimed at enhancing the level of job and life satisfaction and reducing the risk of PBS.

## 2. Materials and Methods

### 2.1. Study Participants

The sample involved 100 oncologic nurses from four Polish hospitals (Lower Silesia region), accounting for approximately 70% of all nurses employed at those institutions. A total of 148 respondents were surveyed. There were 48 questionnaires excluded from further evaluation due to the lack of consent or incorrect completion. The group consisted of 100 certified nurses with different levels of education (high school or university) who had Polish professional licenses. The inclusion criteria were work experience of more than one year in an oncology unit, current work activity as an oncology nurse, and written informed consent to participate in the study. The study was conducted from March 2019 to February 2020. Data were collected using the paper and pencil method. Finally, a group of 100 nurses employed in oncology departments was analyzed. The mean age of the respondents was 43.26 years (SD = 10.69) and the age ranged from 23 to 60 years old. 

### 2.2. Ethical Considerations

The study protocol was approved by the local Bioethics Committee, and all respondents gave written informed consent to participate in the study. The STROBE (The Strengthening the Reporting of Observational Studies in Epidemiology) guidelines were followed due the observational study design. The study was performed in accordance with the Declaration of Helsinki and Good Clinical Practice guidelines.

### 2.3. Research Instruments

#### 2.3.1. Basel Extent of Rationing of Nursing Care—Revised (BERNCA-R)

The BERNCA-R, developed by Schubert et al. [16], was used to assess the RNC when available resources were not adequate to provide sufficient care for all patients. The Polish version of BERNCA-R adapted by Uchmanowicz et al. [17] was used. The quality of nursing services was assessed considering the last seven days, taking into account factors such as the health care system’s cost reduction strategy and declining health care expenditure, the reduction in the number of health care professionals employed, and the reduction in the length of hospitalization (an adverse effect on both the patient and the outcome of nursing care). The questionnaire contains 32 situations in which the rationing of care is necessary, scored on a 4-point Likert scale. The questionnaire score is the average of these 32 questions. It therefore ranges from 0 to 4 and can be interpreted analogously to the interpretation of a single question.

#### 2.3.2. Satisfaction with Job Scale (SWJS)

The SWJS (in Polish—Skala Satysfakcji z Pracy, SSP), developed by Zalewska [18], contains five statements rated by the respondent on a scale of 1–7 points. The SWJS score is the total score of the five questions (range of 5–35 points). Higher scores indicate higher job satisfaction. There are no standard scores for low or high job satisfaction for this tool. The average number of points per question is interpreted according to an established key where: 1—strongly dissatisfied and 7—strongly satisfied. It is a reliable tool for assessing the overall job satisfaction, showing high consistency with similar scales.

#### 2.3.3. Satisfaction with Life Scale (SWLS)

The SWLS was developed by Diener [19] and adapted to the Polish conditions by Juczyński [20]. It consists of five statements rated on a scale of 1–7 points. Each statement relates to a previous life, and the score indicates one’s subjective sense of satisfaction with life. Scores in stens 1–4 indicate a low, scores in stens 5–6 an average, and scores in stens 7–10 indicate a high perception of life satisfaction.

#### 2.3.4. Life Orientation Test-Revised (LOT-R)

The LOT-R is used to measure the level of dispositional optimism. It was developed by Scheier et al. [21] and adapted to the Polish conditions by Poprawa and Juczyński [22]. It contains 10 statements, of which six have a diagnostic value for dispositional optimism. Scores are converted into stens, according to the norms specified in the key to this tool. Scores in stens 1–4 indicate a low level of optimism (i.e., a tendency toward pessimism), scores in stens 5–6 indicate a medium level of optimism (i.e., a neutral attitude), and scores in stens 7–10 indicate a high level of optimism (i.e., a tendency toward optimism).

#### 2.3.5. Maslach Burnout Inventory (MBI)

The MBI was developed by Maslach et al. [23] and adopted in Polish by Pasikowski [24], which allows one to assess the level of PBS in three subscales: emotional exhaustion (EE), depersonalization (DEP), and lack of personal accomplishment (PA). Scores in each of these subscales are expressed on a 0–100 scale (the higher the MBI score, the higher level of PBS). In addition, an overall PBS score was also calculated, which is the average of the three subscales. For the yes/no response versions, there are no standards to determine whether or not the PBS was strong in the respondents [25].

### 2.4. Statistical Analysis

Statistical analysis was performed using the R software 3.6.1 version (R Core Team, Vienna, Austria) [26]. The analysis of quantitative variables was conducted by calculating the mean, standard deviation, median, quartiles, minimum, and maximum. The analysis of the qualitative variables was performed by calculating the number and percentage. The normality of the distribution was performed using the Kolmogorov–Smirnov test. Correlations between the quantitative variables were analyzed using the Pearson’s correlation coefficient (normal distribution) or Spearman’s correlation coefficient (non-normal distribution) for the relationship strengths: |r| ≥ 0.9—very strong, 0.7 ≤ |r| < 0.9—strong relationship, 0.5 ≤ |r| < 0.7—moderate, 0.3 ≤ |r| < 0.5—weak, |r| < 0.3—very weak (negligible) [27]. The normality of the distribution of variables was tested using the Shapiro–Wilk test. The significance level of 0.05 was assumed in the analysis as statistically significant.

## 3. Results

### 3.1. Participants’ Characteristics

All of the study participants were female with a mean age of 43.26 years (SD = 10.69). More than half were in a relationship (86%) and lived in a city (66%). The largest group of respondents (51%) graduated from medical high school, and only 6% had a master’s degree. The average work experience in the study group was 21.25 years (SD = 11.49). Every third of the surveyed nurses completed qualification courses, and every tenth had a specialization in nursing. More than two-thirds of the respondents (83%) worked in one place, all of them were employed under a contract of employment, and their main place of work was inpatient care; the detailed sociodemographic and professional characteristics are present in Table 1.

### 3.2. Results of Rationing of Nursing (BERNCA-R)

The mean BERNCA score in the study group of nurses was 1.55 (SD = 0.15) and the frequency of RNC was between “never” and “rarely” (Table 2). The most frequently rationed tasks (highest mean score) were activation and rehabilitation interventions, administering the prescribed medication or infusion at the right time, reviewing individual patient situations and care plans at the start of the shift, assessing the needs of newly admitted patients, and preparing the patient and family for discharge from the hospital.

### 3.3. Results of Satisfaction with Job (SWJS)

The mean score obtained by the respondents on the SWJS was 11.71 (SD = 5.97), which suggests that the respondents’ answers indicate a state between “dissatisfied” and “rather dissatisfied” with their jobs. Scores ranged from 7 to 18 points, and the median was 11 points (Table 3).

### 3.4. Results of Satisfaction with Life (SWLS)

More than half of the oncology nurses (59%) reported low life satisfaction. In addition, none of the respondents had a high sense of life satisfaction (Table 4).

### 3.5. Results of Life Orientation (LOT-R)

More than half of the oncology nurses (66%) tended to be pessimistic, one in three (31%) showed a neutral orientation, and only three of the respondents showed a tendency to be optimistic (Table 5).

### 3.6. Results of Professional Burnout (MBI)

The mean overall burnout score was 49.27 points (SD = 19.76) out of a possible 100, ranging from 7.41 to 86.67 points. The median score was 52.82, so half of the respondents had occupational burnout at a level lower than 52.82 and half at a level higher than 52.82. The EE was most responsible for job burnout (mean score of 63.56 out of a possible 100 points), lack of PA was less influential (mean score of 47.05), and DEP was the least influential (37.2 points) (Table 6).

### 3.7. Results of Correlations

It was shown that the higher the job satisfaction (SWJS), the more frequent the RNC (BERNCA) (*p* < 0.05) (Table 7). The BERNCA score correlated significantly and negatively with EE, lack of PA, and total MBI score (Table 4). The greater the EE, the stronger the sense of lack of PA, and the higher the PBS (MBI) level, the less frequent the RNC (BERNCA). The other variables did not show statistical significance.

## 4. Discussion

This study attempted to identify factors affecting the RNC among oncology nurses, considering professional burnout, job and life satisfaction, and life orientation. Unfortunately, the results obtained in the study do not allow us to clearly state that any of these factors had an obvious effect on the RNC. However, the comparison of the RNC results with the results of the particular research tools used in this study led to rather surprising conclusions.

The analysis of the obtained results based on the BERNCA questionnaire showed that the mean score for the whole studied group of nurses was 1.55 (SD = 0.15), indicating that the frequency of RNC was in the range between “never” and “rarely”. Similar results were obtained in the study by Uchmanowicz et al. [28,29], Jaworski et al. [30], and Schubert [7], but their studies concerned nurses from other disciplines. Only one study referred to oncology nurses employed in the pediatric hematology and oncology department, in which the authors obtained a score of 2.47 (SD = 0.64), which places the RNC in the range of “sometimes” versus “rarely” [31]. The surprisingly low level of missed care obtained in the present study may indicate a lack of feeling that some aspects of a nurse’s job are being missed, especially if the education level of the nurses surveyed is taken into account (63% had secondary medical education). Most authors investigating the level of rationing of nursing care do not report the degree of variation in the nurses’ education, which is not the same in different education systems. Such differences are particularly evident in Eastern European countries that have undergone restructuring of their education systems. In the labor market, there are both nurses with secondary and higher education [27,32]. Higher levels of unfinished care are reported by nurses from university hospitals, which employ nurses with higher levels of education due to the variety of tasks and higher expectations [33,34].

Based on the obtained results, the most frequently missed nursing care activities include activation and rehabilitation procedures, administering the prescribed medication or infusion at the right time, familiarizing oneself with the individual patient’s situation and care plans at the start of the shift, and assessing the needs of newly admitted patients. In contrast, the most commonly missed areas according to the available literature are conversations with the patient or family [6,35], emotional and psychological support [34,36], and the punctuality of the tasks performed [37,38].

Our study and those of other authors have found that the activities of nurses are most often limited to carrying out medical orders and simple activities to meet the basic needs of the patients. Furthermore, the limitations are most often related to rehabilitation and psychological support. The basis of this situation should be seen in the insufficient number of nursing staff, both in Poland and worldwide [33,39]. These data serve as a basis to direct the attention of those organizing the work of nurses to the difficulties in realizing professional tasks, which can be addressed to improve care in the above-mentioned areas.

The results obtained from the SWJS (SSP) questionnaire showed that the mean score was 11.71 points, indicating a level of job satisfaction between “dissatisfied” and “rather dissatisfied”. The obtained results did not correspond with other studies, in which nurses described their job satisfaction as neither satisfactory nor unsatisfactory [28,30,40,41]. However, there were significant discrepancies between the degrees of job satisfaction in the studies conducted in different countries. For example, nurses generally satisfied with their jobs included Dutch nurses [42].

By correlating job satisfaction with RNC, we found that the higher the job satisfaction (SWJS), the more frequent the RNC (BERNCA). This is quite surprising, because it would seem that people who are dissatisfied with their jobs do not approach the fulfillment of their job duties with adequate commitment [29]. Thus, the hypothesis that higher levels of job satisfaction reduce RNC frequency was not confirmed. However, in the context of the results obtained, it should be mentioned that the strength of the correlation was weak, so the relationship was not significant.

Our study showed that nearly 60% of the oncology nurses scored their life satisfaction as low, while 40% scored it as medium, which may indicate a significant burden on oncology nurses during their professional work, which translates negatively into everyday functioning and reduced life satisfaction. However, most authors indicated that nurses scored life satisfaction as medium [43,44], and in a study by Uchmanowicz et al. [41], nurses and midwives reported high life satisfaction.

Further correlations concerning life satisfaction (SWLS) and life orientation (LOT-R) showed no significant mutual influence. It turned out that these two categories among the respondents did not correlate significantly, so we can surmise that the RNC does not depend on the level of life satisfaction and life orientation. Thus, another hypothesis, which assumed the existence of such a relationship, was not confirmed. Furthermore, these results differ from those reported by other researchers [41,45].

The comparison of the results regarding the level of PBS (MBI) among oncology nurses did not confirm the assumed direction of influence on the RNC levels. Given the relatively high overall PBS and its individual categories (EE, DEP, and PA), there was no significant effect of these factors on the RNC. The relationship of the mentioned categories of burnout (except DEP) was shown, and there was a negative correlation, although its strength was assessed as very weak. Thus, the hypothesis that PBS affects the RNC levels was not confirmed. Of note is also the confirmed existence of PBS among oncology nurses. Although the same factors affecting the level of PBS (age, seniority, number of jobs, number of working hours, etc.) were not evaluated in this study, we confirmed that the average overall score was 49.27/100, which suggests that there was a significant problem in the studied nursing environment.

The issue of RNC is closely related to the quality of nursing care. Activities not performed for various reasons directly affect the level of care. This quality of care depends on the working conditions, type of workload, and tasks performed. The findings of this study also emphasize the validity of the managers’ recognition of the need to improve the professional qualifications, support the implementation of professional tasks, and promote proper organization of work [46]. Ball et al. [47] showed a significant correlation between omitted care and perceived quality of care. However, the lack of time has previously been emphasized as an important factor in RNC [4].

Dabney et al. [48] compared the measurement of missed nursing with the opinions of 729 patients from hospitals in the U.S. about missed care. The lack of the timeliness of tasks performed by the staff and the skill levels were the most commonly indicated. The authors noted a significant correlation between the opinions of the patients and nurses. They also emphasized that conducting research on the topic of RNC should take into account the data from both the patients and staff providing care. Furthermore, Griffiths et al. [49], in their systematic review based on 18 studies, reported on missed care that provided information that 75% or more nurses reported omitting some care. This was related to some work conditions, as low nurse staffing levels were significantly associated with higher reports of missed care and low numbers of registered nurses on staff, which is associated with reports of missed nursing care in hospitals. Missed care is a promising indicator of nurse staffing adequacy.

Continuing research on RNC in Poland is advisable for several reasons. First, they provide a basis for recognizing the deficits in care and allow for the detailed analysis and identification of areas where changes are necessary to maximize the quality of care. Job satisfaction with well-done professional duties determines the overall life satisfaction and is certainly an essential factor in a nurse’s psychological well-being. Obstacles that prevent the delivery of patient care lead to frustration and stress, which in turn affect not only their professional but also their private lives. The results of our study are useful for oncology nurse practitioners, but the findings can also be extrapolated to other nursing settings beyond cancer such as cardiology, internal medicine, surgery, neurology, etc., where PBS can also be a serious phenomenon.

## 5. Conclusions

The findings of this study support the conclusion that: (1) there is a problem with RNC in oncology nurses, but its rate is not high; (2) the RNC depends, to a small extent, on the life and job satisfaction and the level of PBS; (3) the PBS in the studied group of oncology nurses highlights the need for continued research in this area; and (4) more data are needed on the subject of RNC due to the small number of studies in this area.

## Figures and Tables

**Table 1:** Thee sociodemographic characteristics of the study group of oncology nurses (n = 100).

Variable		n	%
Marital status	In a relationship	86	86%
	Single	14	14%
Residence	City	66	66%
	Village	34	34%
Education	Medical high school	51	51%
	Medical school	12	12%
	Bachelor of Science in Nursing	31	31%
	Master of Science in Nursing	6	6%
Work experience [years]	0–5	17	17%
	6–10	7	7%
	11–15	5	5%
	16–20	12	12%
	21–25	16	16%
	26–30	22	22%
	31–35	11	11%
	36–40	10	10%
Postgraduate education	Specialization in nursing	10	10%
	Specialization in organization and management	2	2%
	Qualification courses	28	28%
	Specialist courses	17	17%
Number of work places	One	83	83%
	Two	17	17%
Number of hours spent at work per month	100–180 h	83	83%
	181–230 h	10	10%
	231–300 h	7	7%
Oncology unit	Hematology unit for adults	39	39%
	Hematology unit for children	27	27%
	Hematology outpatient clinic	16	16%
	Bone marrow transplantation unit	18	18%
Work system	Single shift (7 h 35 min)	23	23%
	Day/night shift (12 h)	77	77%
Average number of patients under care during duty	6–10 patients	6	6%
	11–15 patients	18	18%
	16–25 patients	60	60%
	26–35 patients	0	0%
	36 patients and more	16	16%
Number of patients under my care:	Is optimal	1	1%
	It could be bigger	0	0%
	It could be a slightly smaller	61	61%
	It is definitely too big	38	38%

**Table 2:** The analysis of the BERNCA results.

BERNCA [Score]							
N	M	SD	Me	Min	Max	Q1	Q3
100	1.55	0.15	1.53	1.16	1.97	1.46	1.66

**Table 3:** The analysis of the SWJS results.

SWJS [Score]							
N	M	SD	Me	Min	Max	Q1	Q3
100	11.71	2.27	11	7	18	10	13.25

**Table 4:** The analysis of the SWLS results.

SWLS [Score]	Interpretation	n	%
5–17	Low life satisfaction	59	59%
18–23	Moderate life satisfaction	41	41%
24–35	High life satisfaction	0	0%

**Table 5:** The analysis of the LOT-R results.

LOT-R [Score]	Interpretation	n	%
0–12	Pessimistic orientation	66	66%
13–16	Neutral orientation	31	31%
17–24	Optimistic orientation	3	3%

**Table 6:** The analysis of the MBI results.

MBI	N	M	SD	Me	Min	Max	Q1	Q3
EE	100	63.56	25.37	66.67	0	100	55.56	77.78
DEP	100	37.2	24.95	40	0	100	20	60
PA	100	47.05	22.04	50	0	100	37.5	62.5

**Table 7:** The correlations of the BERNCA scores with other outcomes.

Rationing of Care (BERNCA)	Correlation Coefficient	*p*	Direction	Strength
Job satisfaction (SSP)	0.337	0.001	positive	weak
Life satisfaction (SWLS)	−0.147	0.146	---	---
Life orientation (LOT-R)	−0.084	0.405	---	---
Emotional exhaustion (EE MBI)	−0.248	0.013	negative	very weak
Depersonalization (DEP MBI)	−0.175	0.082	---	---
Lack of personal accomplishment (PA MBI)	−0.285	0.004	negative	very weak
Professional burnout (total MBI)	−0.264	0.008	negative	very weak

## Data Availability

The authors confirm that all data underlying the findings described in this manuscript are fully available to all interested researchers upon request.
